# Social Media Influencers, Health Literacy, and Food Literacy: A Correlational Study Among Adolescents

**DOI:** 10.3390/ijerph21121629

**Published:** 2024-12-06

**Authors:** Lies Sercu

**Affiliations:** Department of Linguistics and School of Education, Katholieke Universiteit Leuven (KU Leuven), 3000 Leuven, Belgium; lies.sercu@kuleuven.be

**Keywords:** health literacy, food literacy, adolescent, social media use, correlations

## Abstract

Social media influencers (SMIs) have emerged as a significant alternative source of health-related information for adolescents, apart from their parents, doctors, and schools. It is yet to be determined whether adolescents’ use of social media influencers leads to a deterioration or an improvement of their overall health literacy and food literacy. This study (N = 509), for one thing, investigated adolescents’ health literacy (using the MOHLAA-Q), food literacy (using the SFLQ), and use of social media influencers as sources of information on healthy lifestyles (mapped through self-report questions). For another, it studied whether correlations appear to exist between SMI use, health literacy, and food literacy. The results show that the more frequently adolescents use SMIs as sources of information, the better their health literacy and food literacy are. Consequently, health promotors, such as schools and healthcare professionals, should use the channels adolescents use to enhance their critical health and food literacy, and ultimately their health.

## 1. Introduction

Adolescence is a crucial phase in young people’s development [1]. During this phase of life, some of the most developmentally significant changes in the brain occur in the prefrontal cortex, which is involved in decision-making and cognitive control. Two domains where adolescents need to learn to make their own decisions are nutrition and health. To make healthy choices, adolescents need specific knowledge, attitudes, and skills that have been subsumed under the terms ‘health literacy (HL)’ and ‘food literacy (FL)’. Below, ‘health literacy’ and ‘food literacy’ are described first and a brief survey is provided of the main results obtained so far for adolescents. Next, the introduction looks into adolescents’ sources of information, of which social media (SM) is an important part. Thirdly, linking HL, FL, and SM, this study’s aim is defined.

### 1.1. Adolescents’ Health and Food Literacy

The first domain concerning which adolescents need to make good decisions, namely, health literacy, has been defined differently over the years [2]. In 2000, Nutbeam [3], in his seminal article, conceptualized HL as comprising functional HL, interactive HL, and critical HL. The functional domain refers to basic skills in reading and writing health information, which are important for functioning effectively in everyday life. The interactive domain represents advanced skills that allow individuals to extract health information and derive meaning from different forms of communication. The critical domain represents more advanced skills that can be used to critically evaluate health information and take control of health determinants ([3], p. 2). More recently, Fleary [4] emphasized the critical engagement with health-related (digital) media content as a subskill of HL.

The question of how health-literate European adolescents are was investigated by Paakkari et al. [5]. Their research, based on the Health Behavior in School-Aged Children (HBSC) study, showed that 13.3% of adolescents had low levels of HL, 67.2% medium, and 19.5% high levels. High-level students possess a relatively larger body of knowledge about health issues, can find, understand, and evaluate additional information, and apply that information critically to their lives. Similar results were observed in Flanders. Research by Charafeddine et al. [6] based on the self-report HLS-EU-Q6 showed that 30.8% of Flemish (young) adults possess an insufficient to limited level of HL. Low HL, as measured by the HLS-EU, means that people have difficulties with accessing, understanding, appraising, and using health information. Sercu [7], in a study of adolescents’ HL gained from a performance-based test instrument (e.g., investigating the test takers’ ability to read and understand medicine inserts or nutrition labels), reports that test scores show substantial differences between students in general, technical, and vocational education. The mean test score for HL for pupils in general secondary education is 76.4%, for pupils in technical secondary education 69%, and for students in vocational secondary education a mere 41.9%. Overall, the findings suggest that the HL in particular groups of adolescents (e.g., students in vocational education) is too low, and that there is still room for improvement even in stronger groups.

The second domain concerning which adolescents need to learn how to make healthy decisions is that of nutrition. Just like the research community has made several attempts at presenting a definition of health literacy that reflects its different dimensions, the same is true for food literacy [8,9,10]. A widely-cited definition describes FL as “a collection of inter-related knowledge, skills and behaviors required to plan, manage, select, prepare and eat foods to meet needs and determine food intake. FL is the scaffolding that empowers individuals, households, communities, and nations to protect diet quality through change, and support dietary resilience over time.” ([9], p. 54).

Like HL, FL too has been examined in several European countries, mainly using the Short Food Literacy Questionnaire, developed by Krause et al. [11]. In 2018, in a study among 350 respondents between the ages of 16 and 65, a mean score of 71% was found [11]. In the Polish context, Zwierczyk et al. [12] interviewed 1286 respondents and found a mean sum score of 64%. Trieste et al. [13] applied the SFLQ in Italy to 194 individuals older than 18 years. Their study revealed an average sum score of 71%. Hereby, they concluded that 73% of the respondents were sufficiently nutritionally literate, and 27% were insufficiently nutritionally literate. Another study in Italy, conducted by Palumbo et al. [14], mapped FL using the Italian Food Literacy Survey (FLS-IT). In this study, 5863 individuals older than 18 years participated. Half of the respondents showed problematic FL and one in five was found to have an inadequate level of FL. As is apparent from the above, almost no data are available for adolescent respondents. For Flanders, the Belgian region where the study was reported, so far, no adolescent FL data have been collected.

### 1.2. Adolescents’ Sources of Information on Health and Food Literacy

Whereas in the past, decision-making processes concerning HL and FL were supported by parents, peers, and the school, today, social media, and particularly social media influencers, play a crucial role. The use of social media has increased substantially. “At the time of writing, about 4.59 billion people use social media with many adolescents using their social media accounts across a myriad of applications and platforms.” ([15], p. 1). According to recent statistics, in 2022, individuals spent an average of 151 min on social media each day [16]. A recent APA monitor study [17] indicates that 41% of teens use social media up to 5 hours a day.

One of the pressing questions, internationally, is whether social media use is harmful, especially during adolescence [18,19,20,21,22]. This question is of critical importance, as adolescents may be particularly vulnerable to specific features and advertisements shown to them on social media platforms.

As regards the influence of social media influencers (SMIs), many studies highlight their negative roles as far as HL and FL are concerned [21,22,23,24]. Although SMIs have the potential to disseminate positive public health messages to adolescents who may be difficult to reach through conventional communication channels, influencers have been shown to lack expertise, promote unrealistic body images, unhealthy food and substance use, and provide inaccurate diagnosis and treatment advice. Qutteina et al. [19] found that less than one-third of food-related content highlighted healthy core foods. Unhealthy foods and drinks are excessively showcased, promoted, and endorsed, making up a significant part of the content created by influencers on TikTok, YouTube, and Instagram. Unhealthy food content and advertisements from social media influencers are more memorable and captivating, and they evoke greater pleasure and arousal compared to content from peers, brands, or experts.

Research shows that the relationship between social media use and health literacy, as well as food literacy, is quite intricate and multifaceted, with some authors identifying negative influences, and other authors identifying neutral or positive influences. A study by Levin-Zamir and Bertschi [25] has shown that individuals with higher media health literacy are better at discerning credible health information from misinformation, and may therefore not be affected negatively by the information they receive from social media. Qutteina et al. [26] and Ares et al. [27] show that exposure to food-related content on social media can influence eating behaviors, attitudes, and perceived norms about food. Additionally, social media exposure to food marketing can affect adolescents’ food choices and literacy. For example, exposure to healthy food content can improve food literacy and encourage healthier eating habits, while exposure to unhealthy food marketing can lead to poorer food choices. Overall, social media has the potential to be a powerful tool for improving health and food literacy, but it also requires careful navigation to avoid the pitfalls of misinformation and unhealthy influences.

Against these claims regarding the negative effects of SM on FL and HL, Ferguson et al. [28] posit that caution needs to be issued when attributing, for example, mental health harm to social media use, as the current evidence cannot support this. Montag et al. also report that social media use may have negative consequences for some youth, but positive or no effects for others, suggesting the existence of person-specific effects [15,29,30].

### 1.3. This Study

Together, the above body of research, suggesting mixed results concerning the influence SM may exert on HL and FL, has focused on investigating whether direct effects can be found of SMIs on adolescents’ behavior, yet without taking account of adolescents’ actual levels of health literacy and food literacy. Food and health literacy can be considered person-specific variables that may affect the type of influence SMIs may have on users. The frequency of use of SMIs too can be considered a variable affecting SMIs’ effects.

The mixed-methods study reported here first maps adolescents’ food literacy (FL) and health literacy (HL). Additionally, it investigates to what extent adolescents’ health literacy and food literacy appear to correlate with the frequency of their use of SMIs’ posts. Studying potential correlations will help to answer the question of whether adolescents’ HL and FL correlate positively or negatively with SMI use. Positive correlations would mean that the more adolescents use SMIs as sources of information, the better their HL and FL are. Such a finding would add nuance to earlier findings that tend to point towards only negative influences of SMIs on adolescents’ health behavior. In that sense, this study follows the advice of Powell and Pring [31] to further investigate how the potential positive health impact of social media influencers can be harnessed, while mitigating harmful effects.

## 2. Methods

### 2.1. Sample

A total of 232 boys and 270 girls (509 participants) completed the questionnaire, investigating their FL, HL, and SMI use. The respondents originated from three Flemish catholic schools and one Flemish state school, a division that reflects the Flemish educational landscape well. All participants completed the informed consent form before filling out the questionnaire. An ethical review by the authors’ institution (KU Leuven) was not mandatory at the time the study was conceived and executed. Nevertheless, upon the author’s request, the PRET (Privacy and Ethics Committee KU Leuven) conducted a post hoc assessment (G-2024-8476) of the study in October 2024 and found no ethical issues of concern.

### 2.2. Measurement Instruments

#### 2.2.1. Measurement of Health and Food Literacy

To determine adolescents’ health literacy, this study used the Measurement Of Health Literacy Among Adolescents Questionnaire (MOHLAA-Q) developed by Domanska et al. [32]. This instrument was developed specifically for measuring adolescents’ HL and differs from other instruments, e.g., because of its careful tailoring to the specific health literacies that adolescents possess. Moreover, it is theoretically well-founded and departs from Nutbeam’s [2] model of health literacy, distinguishing between functional HL, interactive HL, and critical HL. Additionally, it uses a mixed-method approach by including health-related knowledge questions as well as self-report questions. In total, the MOHLAA-Q consists of 29 items divided over four scales. Scale A measures the cognitive dimension, specifically, how adolescents interact with health-related information and how easy or difficult they perceive it to find, understand, assess, and apply information related to health care and health promotion. Scale B focuses on adolescents’ communication and interaction skills regarding health information. Scale C addresses the respondent’s attitudes toward his/her health, such as self-awareness, self-control, self-efficacy, motivation, and interest. Finally, scale D measures health knowledge using multiple-choice questions. It has to be remarked that the knowledge items included in the MOHLAA-Q have been debated, arguing that they are not representative enough of what can be expected of adolescents in terms of knowledge about health issues. The number of items is also too small to provide a comprehensive overview of adolescents’ health knowledge.

To determine adolescents’ food literacy, the Short Food Literacy Questionnaire (SFLQ) measurement instrument, developed and validated by Krause et al. [11], was used. The original SFLQ is a 12-item questionnaire to measure a wide range of key skills related to FL, with a focus on individual competencies and abilities required when making healthy food choices. In the original study by Krause et al. [11], the items of the SFLQ were plotted on 4- and 5-point Likert scales, allowing for analyses only at the item level. In this study, it was decided to place the items on a uniform 5-point Likert scale, with identical response categories, thus allowing the calculation of sum scores.

In addition, Krause et al. [11] referenced Nutbeam’s [2] three-part model, but did not apply this classification to the original SFLQ. By contrast, this study opted to categorize the SFLQ items into Nutbeam’s [2] three separate scales. This regrouping resulted in 16 items in total, as 1 item was disaggregated into 5 separate items. Of the 16 items, 10 focused on functional FL (scale A), 1 item focused on interactive FL (scale B), and 5 items measured critical FL (scale C).

Index thresholds for classifying MOHLAA-Q and SFLQ scores into different levels of literacy (see Table 1) were derived from Sørensen et al.’s [33] research. These researchers established threshold values for three levels, namely, limited skills, sufficient skills, and excellent skills.

#### 2.2.2. Measurement of Use of SMI

To explore the relationship between the surveyed students’ HL and FL and their use of social media, the MOOHLA-Q and SFLQ instruments were supplemented with five questions regarding social media use. These items are based on the questions from Digimeter’s annual survey on digital trends in Flanders [34]. Given the health dimension of this study, these more general questions were combined with questions related to the use of social media for health purposes. These questions were selected from the study by Yoon et al. [35], who examined social media use concerning health and food literacy using a questionnaire developed and adapted after a thorough literature review and preliminary testing. The questions we included were: How often do you use social media? How often do you use social media to obtain information regarding a healthy lifestyle? Do you follow SMIs? Do you follow SMI who provide tips regarding a healthy lifestyle? How often do you study the content created by SMIs who provide tips regarding a healthy lifestyle? It was explained that by ‘study’, we meant that the respondent did not simply click on and then click away the content without reading it.

### 2.3. Data Analysis

After data collection, quantitative methods in SPSS 29.0 were used to make statements about 17- and 18-year-old Flemish adolescents’ HL and FL, and their relationship with SMI use. Variables were created to represent mean scores for each MOHLAA-Q and SFLQ scale and a sum score for FL.

Since the MOHLAA-Q had not been previously validated in Flanders, the reliability of each scale was checked using Cronbach’s alpha. The following values were obtained: A (cognitive dimension) 12 items 0.782; B (communication and interaction skills) 4 items 0.567; C (attitudes toward health) 5 items 0.451; D (knowledge) 8 items 0.290. Thus, only scale A (cognitive dimension) was found to have reasonable internal consistency and reliability. Scale B (communication and interaction skills) and scale C (attitude toward health) both failed to reach the required limit of reliability (0.600). This is in line with the original research by Domanska et al. [32], who obtained a Cronbach’s alpha of 0.772, 0.589, and 0.539 for scales A, B, and C, respectively. Despite a poor and unacceptable score, scales B and C were retained in the original study. For scale D (knowledge), internal consistency was measured using Cronbach’s alpha and Kuder–Richardson (KR20) coefficient for dichotomous items (0 = false; 1 = correct). Domanska et al. [32] determined a KR20 of 0.263 for scale D, but accepted this low value as it was to be expected, “because of the intended heterogeneity of the tested knowledge” (p. 14). In this study, Cronbach’s alpha and KR20 were also found to be too low for scale D. Yet, we decided to continue working with scale D. The Cronbach’s alpha indicates that the items do not form a scale. Yet, the items can still provide valid information about the underlying construct, namely, actual health knowledge. Because this body of knowledge is very large and the scale only contained a limited number of knowledge questions, the alpha could not be expected to be (very) high [36,37].

For the SFLQ, a mean score was calculated for each scale and a sum score for the entire questionnaire. Internal consistency and reliability were again tested using Cronbach’s alpha, and the following satisfactory values were obtained: A (functional food literacy) 10 items 0.811; B (interactive food literacy) 1 item—no score was calculated; C (critical food literacy) 5 items 0.779; sum score SFLQ 16 items 0.879.

The normality of the data was tested using the Kolmogorov–Smirnov test and Shapiro and Wilk’s (1965) test. The W value for each scale was >0.96, with the exception of the SFLQ scale B and scale C. A normal distribution for these latter scales was still assumed given the Central Limit Theorem. Consequently, parametric tests, namely, Pearson’s correlation coefficient, independent *t*-test, and ANOVA test were used to analyze our data.

## 3. Results

### 3.1. Health Literacy of Flemish Adolescents

Health literacy was measured using the MOOHLA-Q. As indicated above, scales B and C were not retained due to a lack of internal consistency. The mean score of scale A (12 items) measuring cognitive abilities yielded scores ranging between 2.845 and 2.911 (score range 0–4).

For scale D (8 items), measuring health knowledge, the difficulty index was determined based on the proportion of correct responses. As regards HL, the average difficulty index was 45.9% and varied between 13% and 85%. The obtained result for scale D is lower compared to the study of Domanska et al. [32], since in their study two questions, which were answered correctly by more than 95% of the respondents, were removed from the final questionnaire. Domanska et al.’s [32] study also showed that students overestimated their skills, which was demonstrated by a weak statistical relationship between scale A (cognitive dimension) and scale D (knowledge). This study confirms that there is little to no significant correlation between scales A and D (r = 0.066, N = 509, *p* = 0.139).

An independent *t*-test showed that men in this study group achieved a higher score for scale A (cognitive dimension) (M = 2.959, SD = 3.377) than women (M = 2.816, SD = 0.366), and this difference is significant (t(500) = 4.302, *p* < 0.001). For scale D (knowledge), men also achieve higher scores than women, but the difference is not significant.

### 3.2. Food Literacy of Flemish Adolescents

Food literacy was measured using three scales, each with a response range of 1 to 5. The mean score for scale A (ten items) measured functional food literacy and results ranged between 3.511 and 3.608. Scale B (one item) measured interactive FL with a score ranging from 2.945 to 3.122. The results for scale C (five items), which measured critical FL, ranged from 3.524 to 3.635. Since each scale has a response range of 1 to 5, it was possible to calculate a sum score to further analyze the FL data. The mean sum score ranged from 55.77 to 57.28.

As a Pearson correlation test showed, there is a strong positive relationship (r = 0.714, N = 509, *p* < 0.001) between scale A (functional FL) and scale C (critical FL). A weak positive relationship (r = 0.421, N = 509, *p* < 0.001) exists between scale A (functional FL) and scale B (interactive FL), as well as between scale B (interactive FL) and scale C (critical FL (r = 0.445, N = 509, *p* < 0.001).

An independent *t*-test was also conducted for the SFLQ to determine whether there was a distinction between men and women. The results yield the following picture: men scored higher than women for scale B (interactive FL), scale C (critical FL), and the sum scores, but the difference in each case was not significant. For scale A (functional FL), women achieved a higher score than men, but this difference is also not significant.

### 3.3. Relationship Between Social Media Use and Health and Nutrition Literacy

#### 3.3.1. Descriptive Information About Social Media Use

The questionnaire probed adolescents’ social media use. On a scale from never (0) to daily (4), 97.4% of respondents indicated they use social media daily. In contrast, responses to the question “How often do you use social media to obtain information about a healthy lifestyle?” were divided. The majority rarely (32.8%) use social media to obtain this kind of information, while 30.3% of adolescents do use social media weekly for these purposes. Only 11.8% use social media daily to obtain healthy lifestyle information.

The extent to which adolescents show an interest in following influencers and vloggers who publish online content was also examined. It was found that the majority of the respondents (84.3% or 429) follow influencers or vloggers. Of this group, 53.4% (229 respondents) indicate that they specifically follow influencers or vloggers who provide tips on healthy lifestyles, while 46.6% (200 respondents) indicate that they do not.

#### 3.3.2. Influence of Following Influencers

To examine whether social media use affects adolescents’ health and food literacy levels, an independent *t*-test was conducted to compare two groups. Item 44 (Do you follow influencers or vloggers who post content online?) was chosen as the group variable, and the results of the MOHLAA-Q and the SFLQ were included as test variables. The results of the *t*-test revealed that respondents who do not follow influencers or vloggers achieve lower scores for HL and FL than those who do follow influencers or vloggers, except for scale A (cognitive dimension) of the MOHLAA-Q. Additionally, the difference is significant only for the SFLQ scale A (functional FL) (t(507) = −2.898, *p* = 0.004), SFLQ scale B (interactive FL) (t(507) = −2.724, *p* = 0.007), and the sum score of FL (t(507) = −2.766, *p* = 0.006).

The same *t*-test was conducted with item 45 (Do you follow influencers or vloggers who give tips on healthy lifestyles?) as the group variable. The results of the *t*-test show that respondents who do not follow influencers or vloggers who provide tips on healthy lifestyles achieve lower scores for FL and HL in this study group, except for scale D (knowledge) of the MOHLAA-Q. The difference is significant only for SFLQ scale A (functional FL) (t(427) = −3.581, *p* < 0.001), SFLQ scale B (interactive FL) (t(427) = −2.783, *p* = 0.006), SFLQ scale C (critical FL) (t(427) = −3.335, *p* < 0.001), and the sum score of FL (t(427) = −3.865, *p* < 0.001). For scale A (cognitive dimension) and scale D (knowledge) of the MOHLAA-Q, the difference is not significant.

Overall, the data suggest that respondents with lower use of social media and SMIs show lower levels of FL and HL, yet unclear pictures for critical FL and knowledge (HL) were noted.

#### 3.3.3. Influence of Frequency of Social Media Use

An ANOVA test was first conducted with item 43 (How often do you use social media to obtain information about a healthy lifestyle?) as the independent variable and the results of the MOHLAA-Q and the SFLQ as dependent variables. The results obtained are as follows: MOHLAA-Q: scale A (cognitive dimension) F(4, 504) = 2.583, *p* = 0.036; MOHLAA-Q: scale D (knowledge) F(4, 504) = 2.945, *p* = 0.020; SFLQ: scale A (functional food literacy) F(4, 504) = 13.971, *p* < 0.001; SFLQ: scale B (interactive food literacy) F(4, 504) = 12.759, *p* < 0.001; SFLQ: scale C (critical food literacy) F(4, 504) = 10.360, *p* < 0.001; and SFLQ: sum score F(4, 504) = 16.350, *p* < 0.001.

This test shows with 95% confidence that a significant difference exists between the frequency of using social media to obtain healthy lifestyle information and the outcome achieved on the individual scales of the MOHLAA-Q, the SFLQ, and the sum score of the SFLQ. Significant differences were examined via paired comparison.

This revealed with 95% confidence that adolescents achieve a significantly higher score on scale A (cognitive dimension) of the MOHLAA-Q when they use social media daily (M = 2.996, SD = 0.443) to obtain information about a healthy lifestyle than when they do so more infrequently (M = 2.828, SD = 0.360, *p* = 0.003) or only monthly (M = 2.836, SD = 0.359, *p* = 0.017).

In addition, it was found that adolescents achieve a significantly lower knowledge score (scale D of the MOHLAA-Q) when they never (M = 3.083, SD = 1.476) use social media to obtain information about a healthy lifestyle than when they do so infrequently (M = 3.790, SD = 1.563, *p* = 0.002), monthly (M = 3.877, SD = 1.341, *p* = 0.003), weekly (M = 3.662, SD = 1.397, *p* = 0.011), or daily (M = 3.730, SD = 1.648, *p* = 0.016).

For the SFLQ, the paired comparisons showed that the group that uses social media more frequently consistently scores significantly better than a group that uses social media less frequently for obtaining information about healthy lifestyles. For example, adolescents achieve a significantly higher sum score on FL when they use social media daily (M = 62.032, SD = 8.673) to obtain healthy lifestyle information than when they never do so (M = 51.633, SD = 12.323, *p* < 0.001), rarely (M = 54.790, SD = 7.408, *p* < 0.001), monthly (M = 55.939, SD = 6.905, *p* < 0.001), or weekly (M = 58.312, SD = 8.673, *p* = 0.002).

With 95% confidence, it was found that adolescents achieved a significantly higher score on scale A (cognitive dimension) when they viewed daily (M = 3.049, SD = 0.434) content from influencers or vloggers offering tips on healthy lifestyles than when they did so monthly or less (M = 2.747, SD = 0.3773, *p* < 0.001) or weekly (M = 2.888, SD = 0.346, *p* = 0.008). A significant difference (*p* = 0.025) was also observed with adolescents who watch weekly, scoring higher than adolescents who watch monthly or less.

The results also showed that adolescents achieve a significantly higher score on scale C (critical FL) when they view content from influencers or vloggers that provide healthy lifestyle tips daily (M = 3.895, SD = 0.673) than when they do so monthly or less (M = 3.445, SD = 0.600, *p* < 0.001) or weekly (M = 3.704, SD = 0.486, *p* = 0.036).

## 4. Discussion

This study provides insight into the HL and FL of Flemish adolescents, as well as the relationship between this literacy and their social media use. The degree of literacy in this study is determined using the threshold values and levels (limited, sufficient, excellent) established by Sørensen et al. [33]. The results pertain to Flemish adolescents, living in a well-off country in the center of Europe, with easy access to social media. Yet, they can be considered representative of youngsters in other countries using social media under similar conditions.

Regarding the HL of Flemish adolescents, the results of the MOHLAA-Q should be discussed by scale. Only scales A and D were retained in this study, given the lack of consistency of the other two scales. The obtained mean scores indicate that, based on self-report, adolescents have sufficient cognitive skills related to health, implying that they can find, assess, and apply health-related information to their health. Regarding health knowledge (scale D), the average difficulty index is 45.9%, with a variation between 13% and 85%. This indicates a limited level of health knowledge among Flemish adolescents, which is consistent with the study by Charafeddine et al. [6], who found that nearly one-third of Flemish adults possess an insufficient to limited level of HL, and Sercu [7], who found low levels of health literacy, especially among students in vocational education. It is not surprising in light of findings regarding adults’ health literacy that consistently point out low to sufficient levels in large parts of the population. The findings are also unsurprising because students receive some kind, be it limited, of health education in schools, familiarizing them, for example, with the food pyramid or the necessity to exercise regularly. The findings suggest that health education may lead to cognitive skills (scale A), which is to be applauded, but not necessarily to discrete knowledge (scale D), which is unfortunate. An implication of this is that education should invest more in promoting adolescents’ knowledge about health issues that are relevant to this age group.

In line with Domanska et al. [32], this research shows a weak relationship between scale A (cognitive dimension) and scale D (knowledge). This confirms the findings of previous studies that adolescents tend to overestimate their health literacy. This tendency is seen more often among men. In this study, men generally score higher on HL than women, noting that the difference is significant only for the self-reported cognitive abilities (scale A). This implies that, compared to women, men rate themselves higher on health literacy, but that this does not materialize in higher knowledge. This finding adds nuance to the findings of Charafeddine et al. [6], who found in their study of Flemish adults that men exhibit higher levels of health literacy than women.

The FL of Flemish adolescents was measured using a slightly modified version of the SFLQ, consisting of three scales, each with a response range of 1 to 5. Mean scores for the functional FL (scale A) range from 3.511 to 3.608, indicating adequate functional abilities of Flemish adolescents. For the interactive FL (scale B), the mean scores range from 2.945 to 3.122. This shows limited skills among Flemish adolescents to interactively communicate about food and nutrition. Regarding the critical FL (scale C), mean scores are obtained between 3.524 and 3.635, meaning that Flemish adolescents are sufficiently skilled to deal with food-related information critically and thoughtfully. The average sum score (55.77–57.28, with a maximum of 80) indicates that Flemish adolescents as a whole are sufficiently nutritionally literate. They can find information on healthy nutrition, understand nutrition information leaflets and food labels, are familiar with the Belgian food pyramid and recommendations regarding salt intake and fruit and vegetable consumption, and can compose balanced meals. These mean sum scores are in line with previous research based on the SFLQ in other European countries [11,12,13,14]. In other words, FL, which was defined as a subcomponent of HL, is easier to achieve for adolescents than HL, which is more encompassing. Taken together, these findings suggest that education appears to achieve its knowledge goals, but could support adolescents better in learning how to communicate about food and nutrition-related issues. Communication may enhance critical learning skills and contribute to adolescents’ empowerment.

The relationship between HL, FL, and social media use was also studied. About four in ten Flemish adolescents use social media weekly or more often to obtain information about healthy lifestyles. Interestingly, adolescents who follow influencers who provide tips on healthy lifestyles achieve significantly higher scores for FL (SFLQ: all scales and sum score), especially as regards their functional and critical FL skills. These findings may not come as a surprise, as SMIs who focus on healthy lifestyles will probably devote quite some attention to healthy foods and lifestyles. When adolescents engage with these contents regularly (e.g., daily), they will probably notice and engage with these contents, and apply them to themselves, at least if they deem the information to be trustworthy. These findings corroborate Duplaga’s [38] findings, who found significantly higher HL levels in users of fitness influencer websites.

This study also shows that more frequent use of social media to obtain healthy lifestyle information leads to significantly higher scores on the questionnaires regarding HL and FL, except for health knowledge (MOHLAA-Q: scale D). For scale D, never using social media to obtain healthy lifestyle information leads to significantly lower scores than for the other groups. Moreover, adolescents who daily view content from influencers or vloggers who provide tips on healthy lifestyles achieve significantly higher scores on the HL and FL questionnaires, except for health knowledge (MOHLAA-Q: scale D) and interactive FL (SFLQ: scale B). As regards interactive FL, this finding suggests that adolescents do not necessarily learn to communicate about health issues with other people when following SMIs who provide tips on healthy lifestyles. They may merely process the information for themselves and not interact with others about that information. The finding regarding the MOHLAA-Q, scale D, can be traced back to the comments made earlier on the debatable quality of the items included in scale D.

Overall, the findings go counter to previous studies that point toward the negative effects the use of social media and SMIs may have on youngsters, also in HL- and FL-related areas. For example, as pointed out by Hendriks et al. [39], social media use may lead to more frequent abuse of addictive substances or unhealthy body images. The findings obtained here suggest that at least part of 17–18-year-old adolescents can and do deal critically with the information provided by SMIs, even if there is ample room for improvement given that the HL and FL of many adolescents remain low. Schools could invest more in promoting critical attitudes toward SMIs and SM messages regarding food- and lifestyle-related matters.

## 5. Limitations and Suggestions for Further Research

This research has some limitations, which can be alleviated in future studies. Measuring HL using the MOHLAA-Q has limits since the questionnaire only allows for statements to be made at the level of individual scales and not across the entire questionnaire. Moreover, this study confirms the findings of Domanska et al. [11], namely, that both scale B (communication and interaction skills) and scale C (attitudes toward health) show low internal consistency (Cronbach’s alpha). As a result, the interpretation of the results concerning HL requires a careful approach. In future research, it is advisable to study the MOHLAA-Q in more detail, with specific attention to increasing the internal consistency of scales B and C, for example, by adding or removing items, formulating certain items more clearly, or increasing homogeneity between items. It also seems interesting to investigate the possibilities of calculating a sum score for the MOHLAA-Q, so that general statements about the degree of HL become possible.

For the SFLQ, categorizing the items into three separate scales results in only one item for the interactive FL (scale B), which may raise questions regarding the reliability of this scale since no internal consistency could be measured. Follow-up research may focus on expanding scale B to strengthen its reliability. In addition, it is important to note that the majority of items in the questionnaire administered, both for the MOHLAA-Q and the SFLQ, as well as social media use, rely on self-report and less on knowledge questions. This is to some extent characteristic of the research field; for example, studies on social media use are mainly conducted using self-report measures [40], but it does mean that the results should be interpreted with care. Even if an adequate level in terms of nutritional skills was established, the results could potentially be too positive because of this pure self-report measure. Follow-up research could overcome this, on the one hand by supplementing the questionnaires with knowledge questions, as already partly done in the MOHLAA-Q (scale D), so that the self-report data can be checked against a knowledge measurement. On the other hand, questionnaires could be supplemented with a qualitative dimension, such as interviews and focus groups, to provide insight into adolescents’ attitudes, perceptions, and experiences regarding health and nutrition, thus creating a deeper understanding of the topic and revealing possible reasons for overestimating self-reported data. Additionally, it might be interesting to study the learning trajectories of individual SMI users, investigating what specific information SMIs provide, how that information is processed by the individual, and how, positively or negatively, it affects the individual’s HL and FL.

Analyzing the quality of the information provided by SMIs using thematic analysis techniques, in addition to studying the frequency of use of that information, will shed additional light on the potential effect of SMI use on the development of HL and FL in adolescents. Additionally, the question could be investigated whether there are particular types of engagement strategies or behaviors used by SMIs that are more effective, or what the long-term effects of SMI use are.

In the future, other research samples, originating from other Western or non-Western countries, might be used to study the same research questions. This research will shed light on the representativeness of the current findings for adolescent populations in other nations and areas with other cultural backgrounds.

A final research strand could investigate how educational and healthcare professionals could leverage SMIs to enhance adolescents’ health and food literacy. Since adolescents’ decision-making capabilities are not fully developed, they may need expert guidance in using SMIs for health promotion and maintenance. Evaluation research of educational programs trying to provide this leverage can help to determine how education can best proceed to support learners in making adequate decisions regarding the use or non-use of SMIs to develop their food and health literacy.

## 6. Conclusions and Recommendations

The findings of this study, mainly based on self-reporting, suggest that adolescents have a limited to sufficient level of HL and FL skills, and that their knowledge in these domains is limited. Social media, in particular influencers and vloggers who advise on healthy lifestyles, seem to have a positive impact on adolescents’ FL and HL. Moreover, more frequent use of social media to obtain health information and more frequent viewing of health content from social media influencers seem to be beneficial for their FL and HL, especially as far as the development of functional skills is concerned. These findings run counter to the findings of many previous studies that mainly pointed out the detrimental effects of social media use on adolescents’ HL and FL, but corroborate the findings of studies that have shown the opposite to be true.

Though more research is needed regarding the effect of SMIs on students’ FL and HL, these findings suggest that educators and health professionals might well want to address the students’ use of SMIs for health promotion and maintenance purposes, and assist adolescents in further developing their functional, interactive, and critical health and food literacy skills.

## Figures and Tables

**Table 1:** Threshold values for MOHLAA-Q and SFLQ results.

	Scale	Score Ranges	Limited Literacy	Sufficient Literacy	Excellent Literacy
MOHLAA-Q	A (cognitive)	0–4	0–2.64	>2.64–3.36	>3.36–4
	B (communication and interaction)	0–4	0–2.64	>2.64–3.36	>3.36–4
	C (attitude towards own health)	0–5	0–3.3	>3.3–4.2	>4.2–5
	D (knowledge)	100%	0–66	>66–84	>84–100
SFLQ	A (functional FL)	0–5	0–3.3	>3.3–4.2	>4.2–5
	B (interactive FL)	0–5	0–3.3	>3.3–4.2	>4.2–5
	C (critical FL)	0–5	0–3.3	>3.3–4.2	>4.2–5
	Sum score SFLQ	0–80	0–52.8	>52.8–67.2	>67.2–80

## Data Availability

The raw data supporting the conclusions of this article will be made available by the authors on request.
